# Global trends in *Akkermansia muciniphila* research: A bibliometric visualization

**DOI:** 10.3389/fmicb.2022.1037708

**Published:** 2022-11-10

**Authors:** Zitong Li, Haoran Ke, Ying Wang, Shuze Chen, Xiuying Liu, Qianyun Lin, Pu Wang, Ye Chen

**Affiliations:** ^1^Guangdong Provincial Key Laboratory of Gastroenterology, Department of Gastroenterology, Nanfang Hospital, Southern Medical University, Guangzhou, China; ^2^Hepatology Unit, Department of Infectious Diseases, Nanfang Hospital, Southern Medical University, Guangzhou, China; ^3^Department of Gastroenterology, Beijing Friendship Hospital, Capital Medical University, Beijing, China; ^4^Integrative Microecology Center, Department of Gastroenterology, Shenzhen Hospital, Southern Medical University, Shenzhen, China

**Keywords:** *Akkermansia muciniphila*, gut microbiota, bibliometrics, trends, visualization

## Abstract

**Background:**

*Akkermansia muciniphila* is a member of the gut microbiome, using mucin as sources of carbon, nitrogen, and energy. Since the first discovery of this unique bacterium in 2004, *A. muciniphila* has been extensively studied. It is considered a promising “next-generation beneficial microbe.” The purpose of this paper is to sort out the research status and summarize the hotspots through bibliometric analysis of the publications of *A. muciniphila.*

**Methods:**

The publications about *A. muciniphila* from January 2004 to February 2022 were obtained from the Web of Science Core Collection. Visualization analyses were performed using three bibliometric tools and GraphPad Prism.

**Results:**

A total of 1,478 published documents were analyzed. Annual publication number grew from 1 in 2004 to 336 in 2021, with China being the leading producer (33.36%). De Vos, Willem M was the most productive author with the highest H-index (documents = 56, H-index = 37), followed by Cani, Patrice D (documents = 35, H-index = 25). And Scientific Reports published the most papers. *PNAS* was the keystone taxa in this field, with high betweenness centrality (0.11) and high frequency. The keywords with high frequency in recent years include: oxidative stress, diet, metformin, fecal microbiota transplantation, short-chain fatty acids, polyphenols, microbiota metabolites and so on. The keyword “oxidative stress” was observed to be increasing in frequency recently.

**Conclusion:**

Over time, the scope of the research on the clinical uses of *A. muciniphila* has gradually increased, and was gradually deepened and developed toward a more precise level. *A. muciniphila* is likely to remain a research hotspot in the foreseeable future and may contribute to human health.

## Introduction

*Akkermansia muciniphila*, discovered in 2004, is a Gram-negative, non-motile, ovoid intestinal anaerobe that lacks endospores (Derrien et al., 2004). It belongs to the phylum *Verrucomicrobia* and is the only species of this phylum found in human stools. *A. muciniphila*, which lives in the mucus layer of the intestine, degrades and uses mucin as its sole source of nitrogen, carbon, and energy (Derrien et al., 2004, 2008).

Researchers have investigated “new weapons” at the microbial level to combat disease, and *A. muciniphila* has attracted significant interest in the fields of biological and biomedical research since its discovery. In addition to its relationship with many metabolic diseases (Everard et al., 2013; Depommier et al., 2019; Yan et al., 2021), *A. muciniphila* is negatively associated with numerous conditions including inflammatory bowel disease, amyotrophic lateral sclerosis, autism, epilepsy, and hypertension (Li et al., 2017; Olson et al., 2018; Bárcena et al., 2019; Blacher et al., 2019; Cheng and Xie, 2021; Ke et al., 2021). *A. muciniphila* was implicated in patient responsiveness to programmed cell death protein 1 (PD-1) blockers in cancer immunotherapy studies (Gopalakrishnan et al., 2018; Matson et al., 2018; Routy et al., 2018). Over the past decade, *A. muciniphila* has attracted significant attention in academic circles due to its “probiotic” effect in many diseases; therefore, it is considered a promising “next-generation beneficial microbe” (Cani and de Vos, 2017). An increasing number of studies revealed that *A. muciniphila* plays important roles in various biological aspects; however, the mechanisms underlying its functions remain unclear.

The global trends and hotspots of *A. muciniphila* research have not been studied systematically on a temporal scale despite intensive research interest in recent years. Journal citations and publications can be tracked with bibliometrics through quantitative and qualitative analyses of scientific production and research status (Chen et al., 2014). Therefore, this study aimed to identify the foci and frontiers in *A. muciniphila* research using bibliometric analyses to facilitate further in-depth research at the clinical and basic research levels.

## Materials and methods

### Data collection

**Figure 1:**
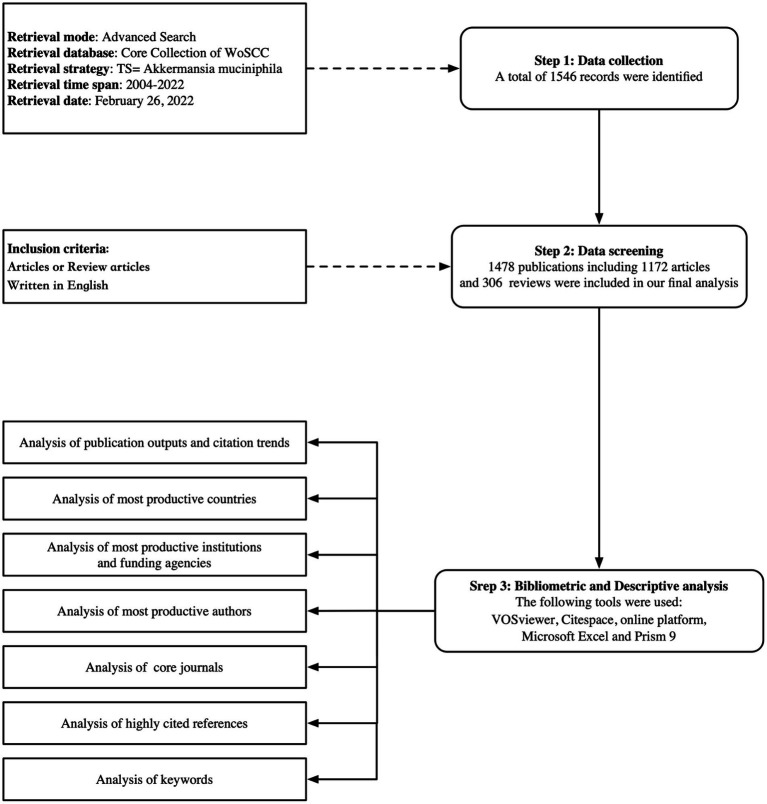
Flowchart of literature filtering and data analysis.

We obtained bibliometric analysis data from the Web of Science Core Collection database (WoSCC), a popular multidisciplinary database in the field of scientometrics (Kokol and Vošner, 2018; Cheng et al., 2022a,b,c). To avoid bias caused by daily database updates, all WoSCC searches were conducted on February 26, 2022. The search formula used was TS = *Akkermansia muciniphila*. In total, 1,546 publications were retrieved, but only 1,478 publications remained after 68 publications were excluded (meeting abstracts, early access, editorial materials, proceedings papers, book chapters, corrections, news items, letters, and/or non-English literature). A plain-text file was exported with all the full records and cited references for further analysis (Figure 1).

Deduplication of the obtained data was performed using the CiteSpace software (version 5.8. R3). Two researchers independently extracted the publications, countries, institutions, funding agencies, authors, journals, citations, keywords, highly cited references, Hirsch index (H-index; Engqvist and Frommen, 2008), and average citations per item (ACI). To ensure data accuracy and reliability, discrepancies were reconciled *via* discussions and negotiations. The 2021 Journal Citation Report (Clarivate Analytics, Philadelphia, PA, United States) was used to obtain journal information.

### Data analyses

**Figure 2:**
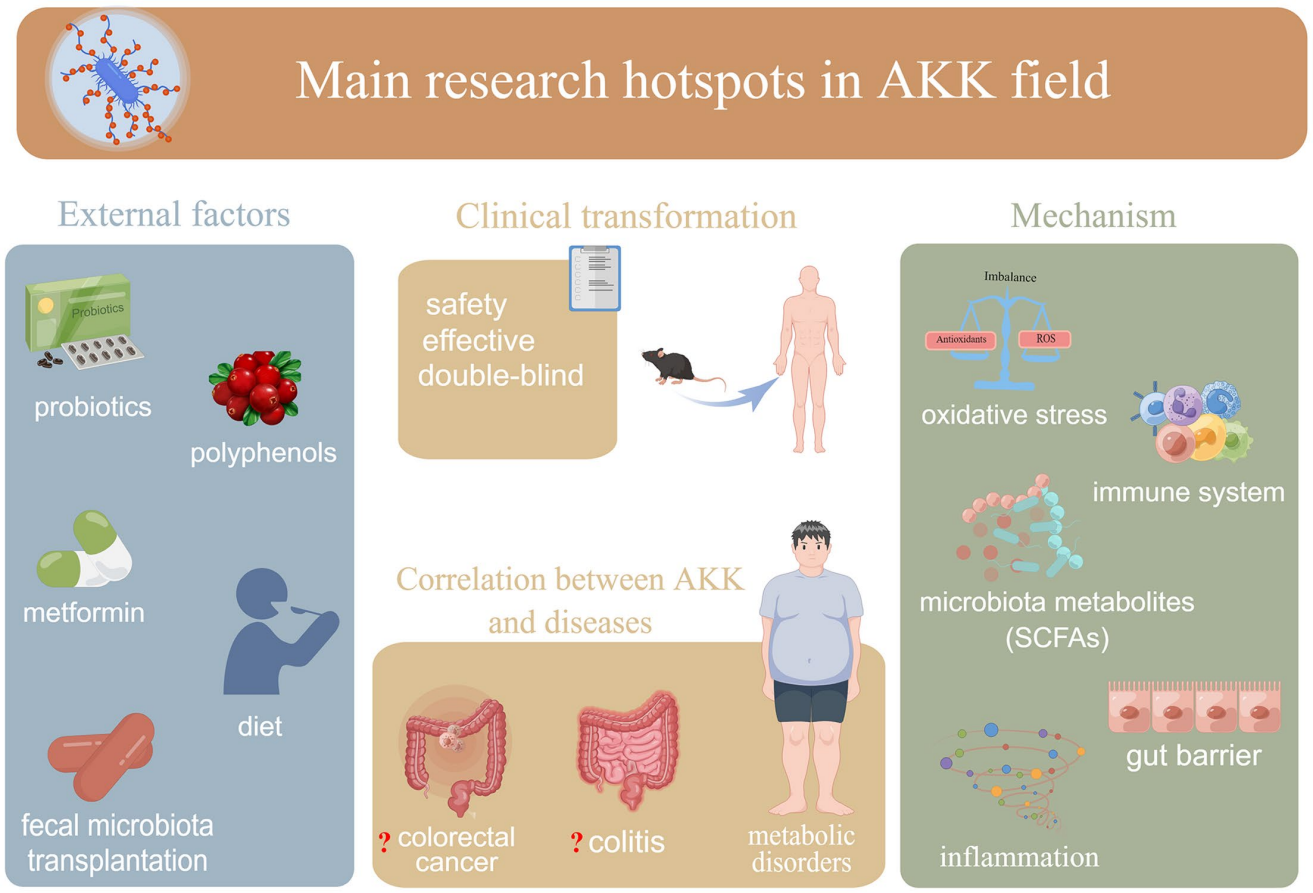
Recent hotspot directions of *A. muciniphila* research: (i) external factors affecting *A. muciniphila;* (ii) mechanisms underlying the association between *A. muciniphila* and hosts (including bacteria); (iii) correlations between *A. muciniphila* and different diseases; (iv) safety and efficacy of clinical use of *A. muciniphila.* AKK:*A. muciniphila*.

The CiteSpace (version 5.8. R3; Chen, 2004; Chen et al., 2014), VOSviewer (van Eck and Waltman, 2010), an online bibliometric platform,^1^ and GraphPad Prism (version 8.4.3) were used for bibliometric and visual analyses. The relevant information was summarized in a table using Microsoft Excel (version 16.58). Figure 2 is drawn with Figdraw.^2^

## Results

### Publication and citation trends

**Figure 3:**
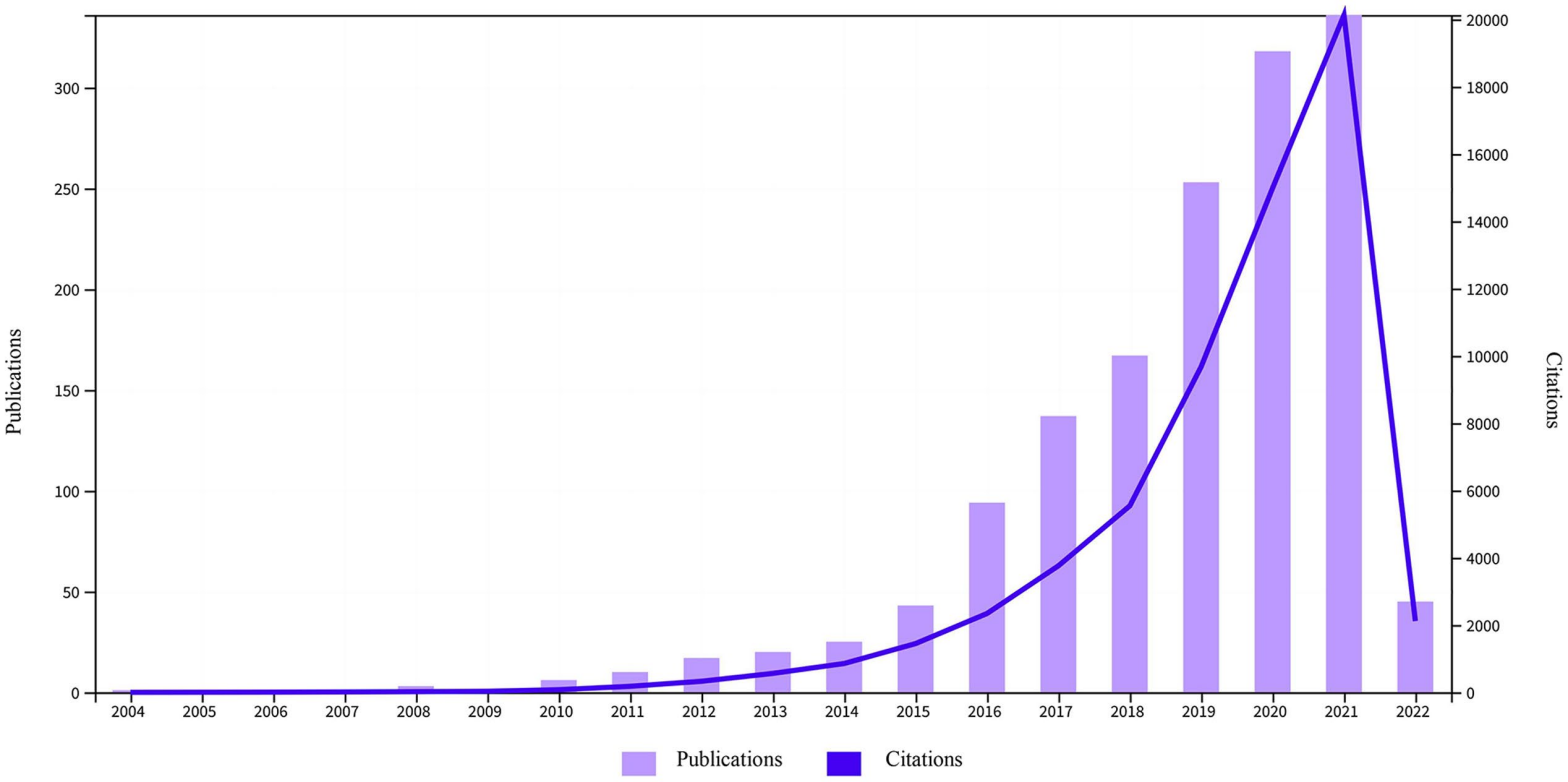
Trends in publications and citations regarding *A. muciniphila* (2004–2022).

In total, 1,478 papers (1,172 original articles and 306 reviews) were analyzed (Figure 1). Figure 3 shows the upward trend in publications and citations over the past 18 years. The number of publications rose from one to 336 from 2004 to 2021. Approximately 88.30% of the articles were published between 2016 and 2021, and the number of publications in the first 2 months of 2022 exceeded that in all of 2015. The total number of citations was 62,095 (51,188 if excluding self-citations).

### Analysis of the countries/regions

**Figure 4:**
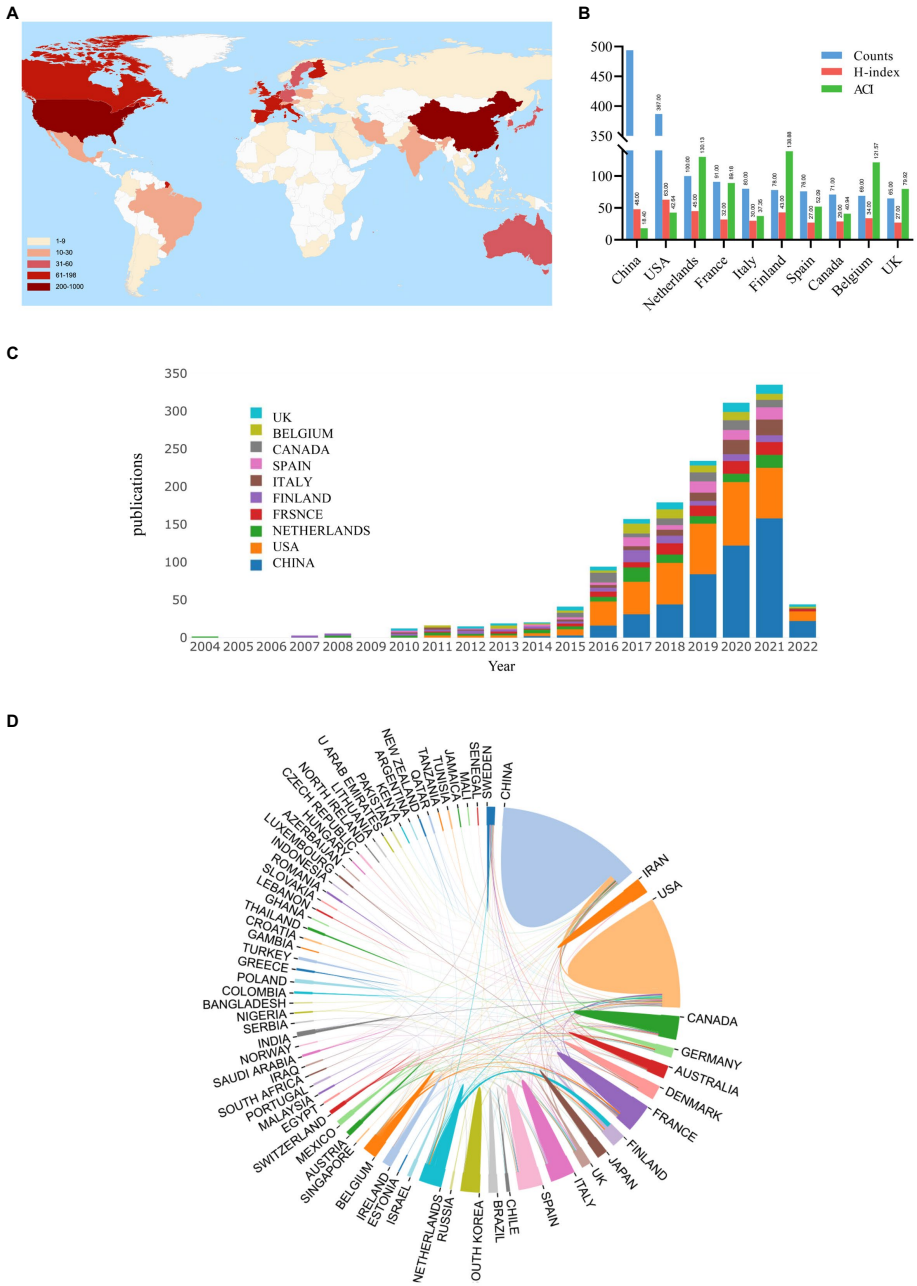
Analysis of the contribution of different countries/regions to *A. muciniphila* research. **(A)** Geovisualization of *A. muciniphila* research distribution. Color shades correlate with the number of articles published. England, Northern Ireland, Scotland, and Wales were reclassified together as the United Kingdom; Taiwan was merged into China. **(B)** The publication counts, H-index, and ACI of the top 10 most productive countries/regions. **(C)** Trends in *A. muciniphila* publications from the top 10 countries/regions from 2004 to 2022. The colors represent different countries/regions. **(D)** Cooperation of countries/regions involved in *A. muciniphila* research. The proportion of the area correlates to the number of national publications, and the thickness of the line reflects the strength of cooperation between countries.

Figure 4 shows the distribution of *A. muciniphila*-related publications worldwide. East Asia, North America, Western Europe, and South Europe were the most productive countries/regions (Figure 4A). Figure 4B and Figure 4C demonstrate the basic information and trends in annual publication output, respectively, among the top ten countries (2004–2022). Seventy-three countries/regions produced publications on *A. muciniphila*. China ranked first with 493/1,478 publications (33.36%), followed by the United States (387/1,478; 26.18%). The United States had the highest H-index (63), whereas Finland (138.88) and the Netherlands (130.13) had the highest ACI (Figure 4B). In addition, network analysis was used to identify cooperative relationships between countries. As shown in Figure 4D, the closest cooperation occurred between China and the US, followed by that between Finland and the Netherlands.

### Analysis of the institutions and funding agencies

**Figure 5:**
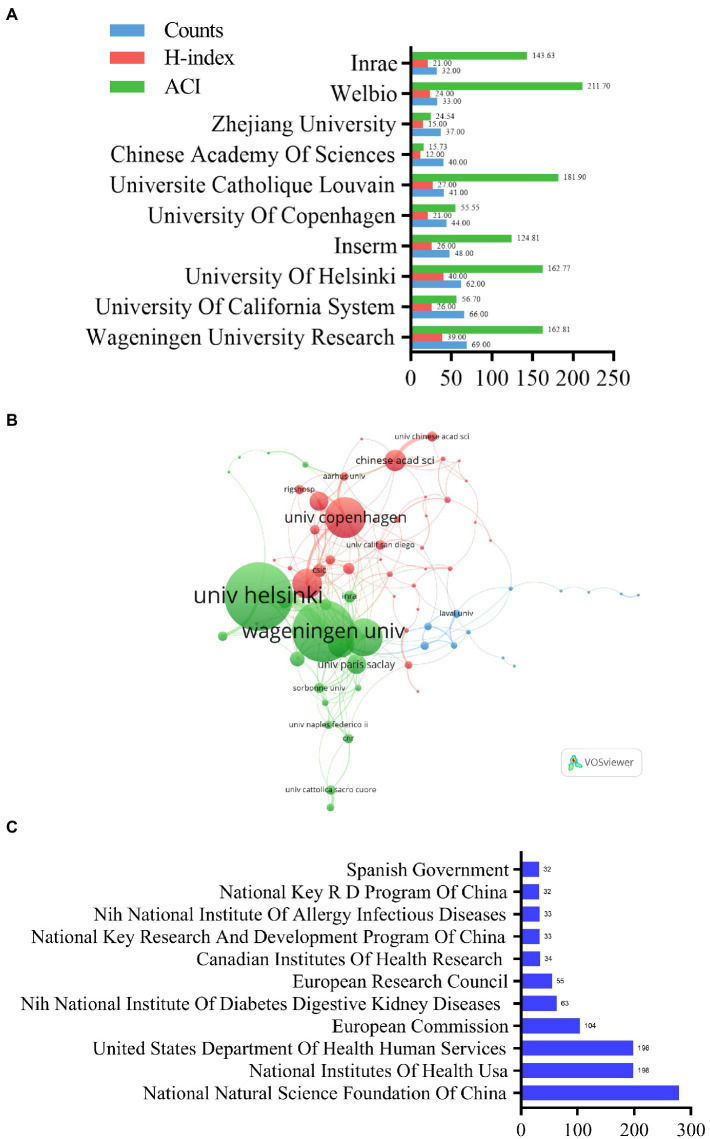
Analysis of the contribution of different institutions and funding agencies to *A. muciniphila* research. **(A)** The publication counts, H-index, and ACI of the top 10 most productive institutions. **(B)** Co-authorship analysis of the institutions. Each node represents a different institution. Node size reflects the strength of cooperation. **(C)** Top 10 related funding agencies which support *A. muciniphila* research (Spanish Government and National Key RD Program of China tied for 10th).

Of the top 10 institutions, Wageningen University & Research in the Netherlands had the high H-index and was the most productive institution (H-index = 39, publications = 69; Figure 5A). It was followed by two institutions each from France, Belgium, and China, and one each from the United States, Finland, and Denmark (Figure 5A). Walloon Excellence in Life Sciences and Biotechnology (WELBIO) had the highest ACI (211.70). Figure 5B illustrates the collaborations between institutions with a minimum number of eight published articles (associated institutions only). The node size indicates the degree of cooperation of an institution with other institutions (weighted by the total link strength [TLS]; the higher the TLS value, the stronger the cooperation strength; Li et al., 2022). A total of 201 lines and 70 nodes were present on the institutional network map, and the University of Helsinki (TLS = 79) and Wageningen University Research (TLS = 72) had the highest TLS (Figure 5B).

The funding agencies’ contributions showed similar trends as that of the countries/region (Figure 5C). Four agencies in the US and three in China were included. The National Natural Science Foundation of China was the largest sponsor (279 studies; Figure 5C), followed by the National Institutes of Health (United States) and the United States Department of Health and Human Services.

### Analysis of the most productive authors

**Figure 6:**
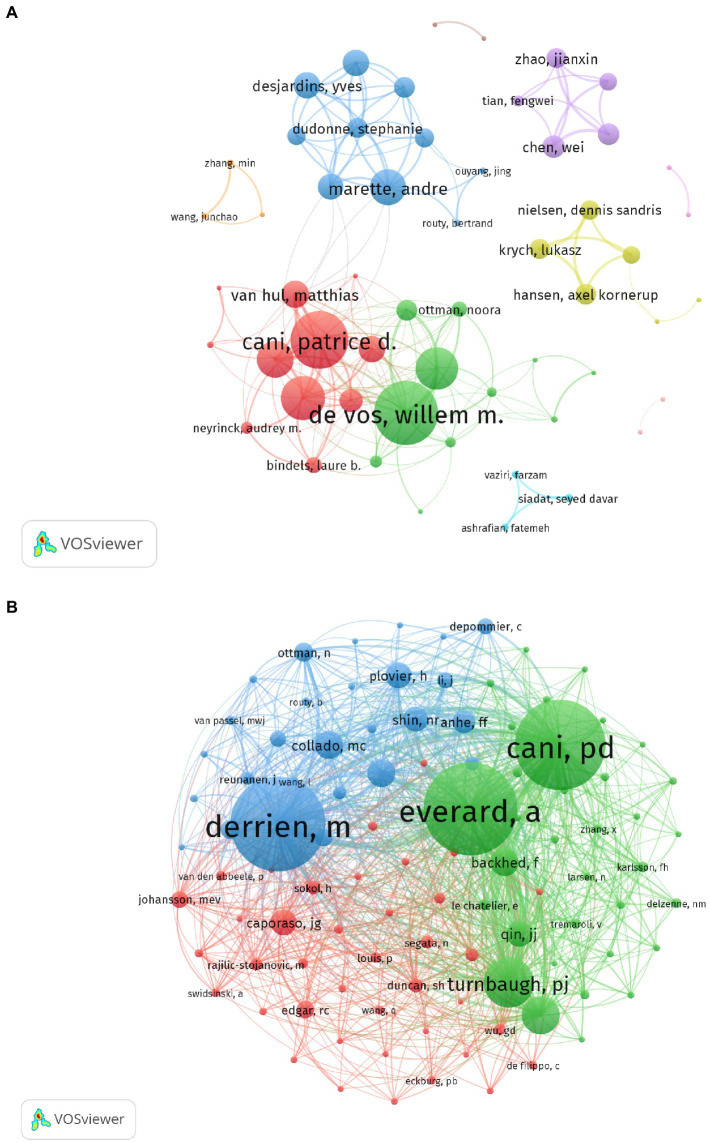
Contribution analysis of authorship in *A. muciniphila* research. **(A)** Co-authorship analysis of authors. Each node represents a different author. After the network was generated, cooperation between authors was shown as same-colored clusters. The size of the nodes reflects the strength of their cooperation. **(B)** Co-citation analysis of cited authors generated by VOS viewer. Each node represents a different cited author. The size of the nodes is weighted by citations.

The most productive authors (those with at least 15 publications) are listed in Table 1. De Vos, Willem M. from the University of Helsinki and Wageningen University was the most prolific author with the highest H-index (publications = 56, H-index = 37), followed by Cani, Patrice D. from the Universite Catholique de Louvain (publications = 35, H-index = 25). Everard, Amandine from the Universite Catholique de Louvain had the highest ACI (344.25). Figure 6A illustrates the collaboration network of authors who had at least six publications (weighted using TLS). The network consisted of 61 nodes and 174 lines (six nodes not shown). De Vos and Willem had the highest TLS (111), followed by Cani and Patrice (100) and Delzenne and Nathalie (77). The co-citation analysis included 101 nodes and 4,999 lines for authors with 70 citations or more. Derrien, Muriel (citations = 1,153), Everard, Amadine (citations = 1,126), and Cani and Patrice (citations = 1,096) were the most-cited authors (Figure 6B).

**Table 1:** The most productive authors (those with at least 15 publications) in the field of *A. muciniphila* research.

**Rank**	**Author (Country)**	**Counts**	**% of 1,478**	**Institutions**	**H-index**	**ACI** ^**a**^
1	De Vos, Willem M (Netherlands; Finland)	56	3.79	Wageningen University University of Helsinki	37	168.20
2	Cani, Patrice D (Belgium)	35	2.37	Universite Catholique de Louvain	25	200.14
3	Belzer, Clara (Netherlands)	32	2.17	Wageningen University & Research	21	165.38
4	Chen, Wei(China)	19	1.29	Jiangnan University	10	20.84
5	Delzenne, Nathalie M (Belgium)	19	1.29	Universite Catholique de Louvain	14	226.16
6	Hansen, Axel Kornerup (Denmark)	18	1.22	University of Copenhagen	11	35.61
7	Everard, Amandine (Belgium)	16	1.08	Universite Catholique de Louvain	13	344.25
8	Marette, Andre(Canada)	16	1.08	Laval University	13	71.31
9	Nielsen, Dennis Sandris (Denmark)	15	1.01	University of Copenhagen	10	42.60
10	Siadat, Seyed Davar (Iran)	15	1.01	Pasteur Institute of Iran	5	6.33
11	Zhang, Hao (China)	15	1.01	Jiangnan University	9	14.80

a ACI: average citations per item.

### Analysis of the journals

**Figure 7:**
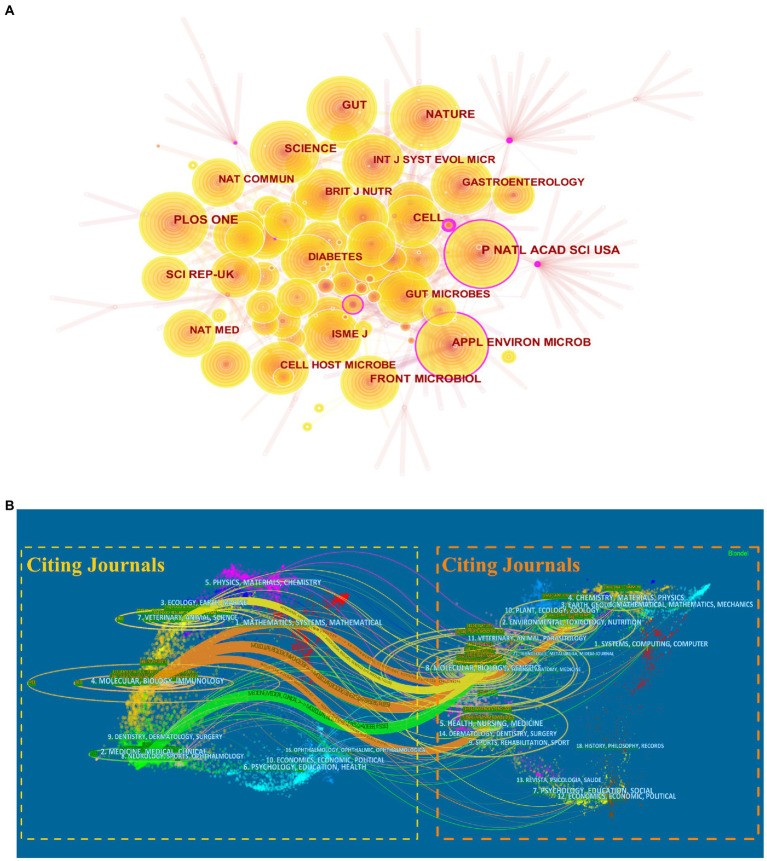
Analysis of core journals of *A. muciniphila* research. **(A)** Co-citation analysis of journals. Each node represents a different journal. The size of the nodes is weighted by the number of citations. The purple outer circle highlights nodes with intermediary centrality greater than 0.1. **(B)** Dual-map overlay of the journals publishing *A. muciniphila* articles generated by CiteSpace. Each label indicates a separate research subject covered by the journal. On the map, the left side represents the citing journals, while the right side represents the cited journals. There are different colored lines for the different reference paths, which begin with the citing map and end at the cited map.

The top 10 most productive journals in *A. muciniphila* research are listed in Table 2, and accounted for approximately 24.70% of all publications (365/1,478). *Scientific Reports* published the highest number of papers (61/1,478), followed by *Nutrients* (59/1,478) and *Frontiers in Microbiology* (54/1,478). A co-citation analysis was also conducted to investigate the influence of the journals. The top four most-cited journals were *Proceedings of the National Academy of Sciences of the United States of America* (PNAS; 1,181), *PLOS One* (1,129), *Nature* (1,092), and *Gut* (1,092; Figure 7A). Notably, PNAS had a central value of 0.11, indicating a high betweenness centrality.

**Table 2:** Top 10 journals for *A. muciniphila* research.

**Rank**	**Journal**	**Counts**	**% of 1,478**	**IF (2021)**	**JCR (2021)**	**H-index**	**ACI** ^**a**^
1	*Scientific Reports*	61	4.13	4.996	Q2	26	36.33
2	*Nutrients*	59	3.99	6.706	Q1	21	22
3	*Frontiers in Microbiology*	54	3.65	6.064	Q1	26	46.46
4	*Food Function*	33	2.23	6.317	Q1	15	16.73
5	*Gut Microbes*	33	2.23	9.434	Q1	13	34.45
6	*Plos One*	30	2.03	3.752	Q2	19	75.17
7	*Molecular Nutrition Food Research*	26	1.76	6.575	Q1	14	26.27
8	*Microorganisms*	25	1.69	4.926	Q2	10	35.56
9	*Gut*	24	1.62	31.793	Q1	22	234.58
10	*Frontiers in Cellular and Infection Microbiology*	20	1.35	6.073	Q1	7	17.3

a ACI: average citations per item.

Figure 7B shows the dual-map overlay depicting the flow from the citing to cited subject categories, mainly including three orange pathways (from “molecular, biology, immunology” to “molecular, biology, genetics,” “environmental, toxicology, nutrition,” and “health, nursing, medicine”), one green pathway (from “medicine, medical, clinical” to “molecular, biology, genetics”), and one yellow pathway (from “veterinary, animal, science” to “molecular, biology, genetics”).

### Analysis of highly cited references

Supplementary Table S1 lists the top 10 co-cited articles on *A. muciniphila* research, which are generally viewed as the ‘classics’ (Li et al., 2022). The most-cited paper was published by Everard et al. (2013) in PNAS, and was cited 319 times in this field. Dao et al. (2016) and Derrien et al. (2017) published the second and third most-cited papers, respectively.

**Figure 8:**
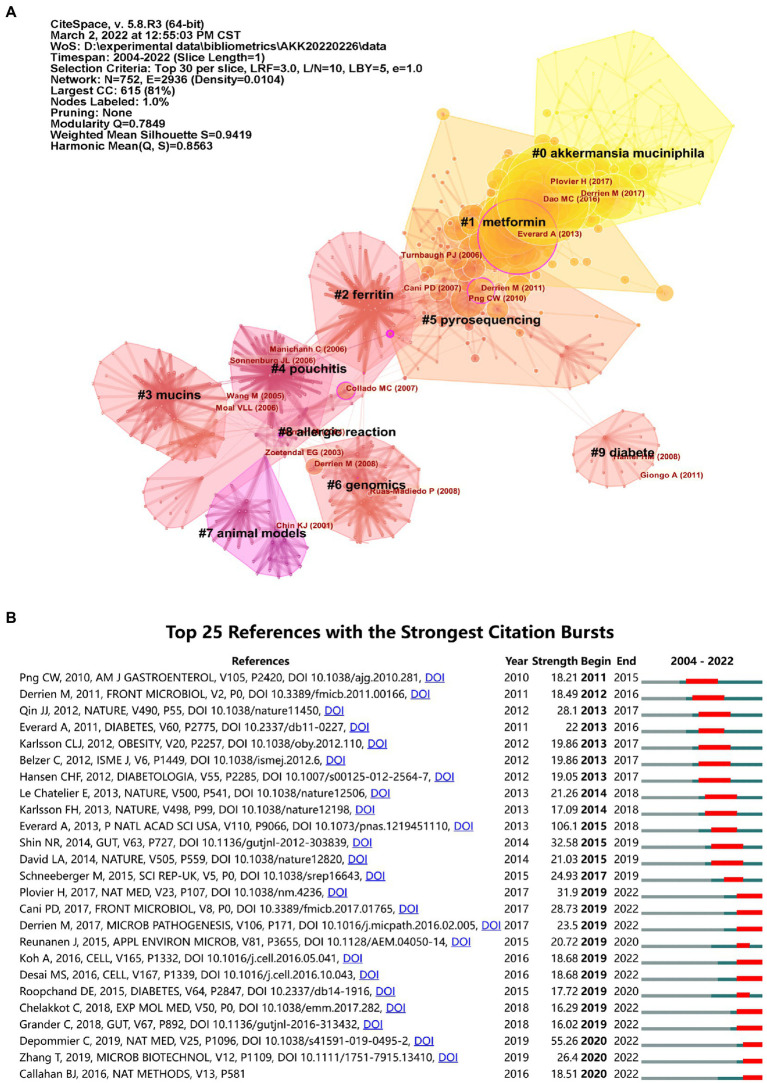
Analysis of references in *A. muciniphila* research. **(A)** The network map of co-cited references. Each node represents a different reference. The cited references form several natural clusters, which are closely related. The purple outer circle highlights nodes with intermediary centrality greater than 0.1. **(B)** Top 25 *A. muciniphila*-related references with the strongest citation bursts (2004–2022).

Research hotspots were traced using co-citation analysis of the references. The co-citations were visualized and clustered to analyze the research focus. The modularity value (Q-value) and the mean silhouette value (S-value) were calculated to evaluate the clustering quality, where Q > 0.3 and S > 0.7 indicate that the clustering structure is significant and convincing (Wu et al., 2021a). Figure 8A illustrates the top 10 largest clusters with good homogeneity (S = 0.9419, Q = 0.7849). Citation bursts were mainly concentrated in cluster #0 (*A. muciniphila*) and cluster #1 (metformin). As shown in Figure 8B, the reference with the highest citation burst strength was by Everard et al. (2013). Notably, bursts in several studies have been increasing recently (Callahan et al., 2016; Desai et al., 2016; Koh et al., 2016; Cani and de Vos, 2017; Derrien et al., 2017; Plovier et al., 2017; Chelakkot et al., 2018; Grander et al., 2018; Depommier et al., 2019; Zhang et al., 2019).

### Analysis of the keyword research knowledge

**Figure 9:**
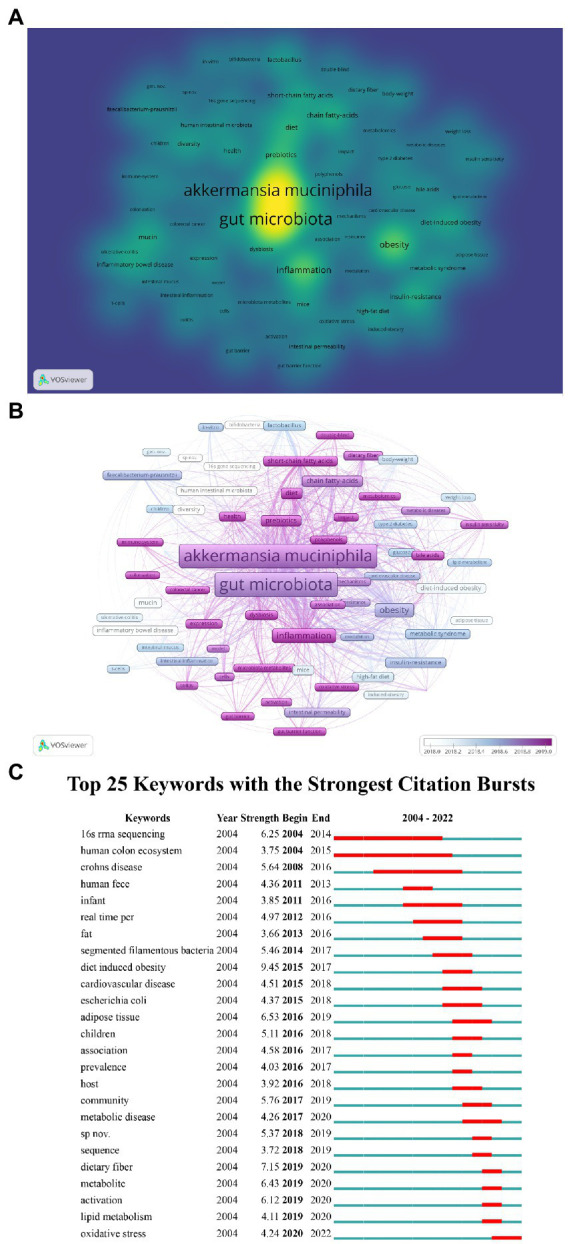
Author keywords analysis. **(A)** Keywords co-occurrence heatmap. The heatmap color and size reflect the frequency of keywords. **(B)** Overlay visualization of *A. muciniphila*-related keywords. Each node represents a different author keyword. The color of the nodes corresponds to the average year in which the keyword appeared according to the color gradient shown at the bottom right. **(C)** Top 25 *A. muciniphila-*related keywords with the strongest citation bursts (2004–2022).

After synonym merging, 2,389 author keywords were obtained from 1,478 articles. A heatmap was generated for author keywords that occurred at least 30 times from 2004 to 2022 in the *A. muciniphila* research field (Figure 9A). The heatmap is colored and sized according to the frequency of the keywords. The top 10 keywords were “*A. muciniphila*,” “gut microbiota,” “obesity,” “inflammation,” “prebiotics,” “chain fatty acids,” “diet,” “insulin resistance,” “metabolism,” and “diet-induced obesity.” Additionally, Figure 9B color-codes the keywords based on the year in which they appeared. The keywords with an average appearance year after 2019 (more recent appearance) include “microbiota metabolites,” “metformin,” “fecal microbiota transplantation,” “oxidative stress,” “immune system,” “short-chain fatty acids,” “diet,” “colorectal cancer,” “diabetes,” “gut barrier function,” “double-blind,” “polyphenols,” etc. (Figure 9B). Furthermore, among the top 25 keywords with the strongest citation bursts, “oxidative stress” was observed to be increasing in frequency recently (Figure 9C). There may be a continued focus on these emerging keywords in the future.

## Discussion

The frequency of *A. muciniphila* publications has shown exponential growth curve over the last 18 years, possibly due to growing treatment needs and advances in microbiome technology (e.g., 16S rRNA sequencing, metagenomics, metabolomics, etc.). The remarkable efficacy of *A. muciniphila* on obesity and diabetes has promoted its exploration in various fields. The cliff-like shape of the citation curve shown in Figure 3 indicates that this field is a research hotspot, and its popularity will continuously increase and become a hot research topic in the future.

The most productive countries were China and the United States. Initially, the United States was the topmost productive country; however, with increasing interest in the field among Chinese researchers, this gap gradually narrowed as publications from China increased in frequency. Combined with the institutional and funding agencies analyses, the high output of China and the United States is likely related to human investments and financial resources. The H-index is a crucial parameter for assessing the publication quality and academic influence of countries, institutions, journals, or researchers (Engqvist and Frommen, 2008). As with the H-index, the ACI can also represent the scientific output and academic status of publications. Based on the ACI, Finland played a crucial role in this field. Although China had the highest number of publications, the two institutions from China had the lowest average citation rate and H-index among the top ten institutions. Thus, the quality of the publications requires improvement. Cooperation between countries is essential, as strong cooperative relationships were observed among the countries with the most publications and highest ACI. Many countries/institutions have low influence levels, and inter-agency cooperation should be prioritized.

Analysis of the cooperative relationships between authors revealed inter-author connection networks. De Vos and Willem was the most productive author with the highest H-index. The author and the institutions and/or country the author belongs to can exert a significant influence on the emerging *A. muciniphila* research field. Furthermore, De Vos and Willem is a leading expert in gut microbiota research and is at the forefront of exploring microorganisms through molecular (meta-) genomics and systems approaches, focusing on the human gut (Belzer and de Vos, 2012). Another highly influential author is Cani and Patrice. Their research interests include interactions among gut microbes, the host, and specific biological systems, such as the endocannabinoid and the innate immune system, and their associations with metabolic disorders. Interestingly, De Vos and Willem and Patrice and Cani are co-founders of A-Mansia Biotech SA, the *Akkermansia* company.^3^ They facilitate the transformation of basic research into clinical applications (Depommier et al., 2019). It is evident that their academic collaboration has contributed to their current success in this field. Both of these pioneering researchers make significant contributions to this field, and many highly cited references and citation burst references were published by their teams (Everard et al., 2011, 2013; Belzer and de Vos, 2012; Schneeberger et al., 2015; Cani and de Vos, 2017; Derrien et al., 2017; Plovier et al., 2017; Depommier et al., 2019).

Journal statistics help researchers select suitable journals for publishing their research. *Scientific Reports*, *Nutrients*, and *Frontiers in Microbiology* were the major journals that published *A. muciniphila-*related articles. The betweenness centrality of nodes in a network is a vital centrality indicator (Wu et al., 2021b), indicating co-citation relationships between multiple nodes and which journals are “transportation hubs.” *PNAS* had high betweenness centrality and frequency, and is considered a keystone taxon in this field. As article carriers, journals reflect the position of published articles in the field. The key position of *PNAS* is the result of the articles published in the field, such as the study published by Everard et al. (Everard et al., 2013), which is discussed in the next paragraph. These journals are predicted to publish more high-quality research. A dual-journal overlay shows how topics and journals are interrelated (Li et al., 2022). Although this research draws on the distribution of the cited articles, citing articles on *A. muciniphila* were more active in the “veterinary, animal, science,” “molecular, biological, immunology,” and “medicine, medical, genetics” fields.

Within *A. muciniphila* research, highly cited references or references with strong citation bursts are important nodes through which *A. muciniphila* has distinguished itself from other probiotics and has become a “next generation probiotic.” Citations are a simple and effective indicator of the impact and quality of research. The article published in *PNAS* by Everard et al. (2013) has the highest citation frequency as well as a high mediation centrality, indicating that its content provides information on a currently relevant topic. This article proposes that live *A. muciniphila* can reverse high fat diet-induced metabolic disorder in mice by restoring mucus secretion and improving intestinal permeability, while heat-killed *A. muciniphila* lacks this effect. The article details an important milestone in our knowledge on the interaction between microbiota and the intestinal epithelium. The second most-cited article is a clinical research study published by Dao et al. (2016). The authors described the relationship between *A. muciniphila* and metabolism in overweight/obese adults after clinical dietary intervention. The article stated that *A. muciniphila* may be a potential prognostic tool for predicting the success of dietary interventions. The third most-cited paper was published by Plovier et al. (2017). This study identified a component of the *A. muciniphila* outer membrane protein-AMUC_1100 (which interacts with TLR2) that plays a role in reducing fat development, insulin resistance, and dyslipidemia in mice after pasteurization. This study proposed a solution for the unknown safety of *A. muciniphila* growth medium substances when ingested by humans. The most logical explanation for a sudden increase in the citation frequency of an article is that it addresses a specific lack of information in currently available literature (2003). The article with the strongest citation burst was the article published by Everard et al. (2013). The second-strongest citation burst was the randomized double-blind controlled study published by Depommier et al. (2019), which detailed the first human experimental results for *A. muciniphila* supplementation. This study indicated that *A. muciniphila* is safe and well-tolerated by patients; it also suggested that dead *A. muciniphila* bacteria may be more beneficial than live bacteria. The third strongest citation burst was that of an article published by Shin et al. (2014), which determined whether the antidiabetic effects of metformin were associated with changes in gut microbiota composition. It is evident from the changes in citation burst trends that the research hotspot had transitioned from the correlation between *A. muciniphila* and disease to the causal relationships between them, and from animal experiments to human studies of safety and efficacy.

Since keywords represent a publication’s core content, keyword co-occurrence analysis (a method developed through bibliometric research and data visualization) can be applied to identify popular research topics in a particular field at a certain time. Our results showed the following four main research directions in the *A. muciniphila* field (Figure 2):

- External factors affecting *A. muciniphila* (e.g., “diet,” “polyphenols,” “metformin,” and “fecal microbiota transplantation”). In recent years, dietary strategies for improving gut *A. muciniphila* abundance have attracted research and development interest (Zhou, 2017). Although these promotion strategies are not necessarily applicable to the general population, these results strongly suggest the potential efficacy of certain foods or supplements for increasing intestinal *A. muciniphila* levels.
- The correlation between *A. muciniphila* and different diseases. The associations between *A. muciniphila* and metabolic disorders, including obesity, type 2 diabetes, nonalcoholic fatty liver disease, and cardiovascular diseases, are key to explaining many existing questions in *A. muciniphila* research. Highly cited references or references with strong citation bursts with respect to *A. muciniphila* research have all been related to metabolic disorders. For specific developmental milestones in research on *A. muciniphila* and metabolic disorders, please refer to the earlier discussion on references. In recent years, colitis and colorectal cancers have also attracted much attention. This could be attributed to the still-unclear relationship between *A. muciniphila* and colitis or colorectal cancers. Ring et al. (2019) demonstrated that *A. muciniphila* colonization does not affect colitis. This is somewhat different from the conclusions of an earlier study by Seregin et al., which reported that *A. muciniphila* can promote the occurrence of colitis in mouse models. Some studies have found that the abundance of *A. muciniphila* is increased in patients with colorectal cancer (Sanapareddy et al., 2012; Weir et al., 2013; Zackular et al., 2013; Dingemanse et al., 2015; Wang et al., 2022), whereas others have suggested that *A. muciniphila* is unrelated to colon tumors (Lopez-Siles et al., 2018) or even prevents colitis-associated colorectal cancer (Wang et al., 2020). Thus, the relationship between *A. muciniphila* and both diseases remains controversial. However, it is worth noting that most of the current studies have not distinguished between these microorganisms to the species level. To clarify the relationships between *A. muciniphila* and various diseases, this nuance should not be ignored in future clinical studies.
- Mechanisms underlying *A. muciniphila*–host (including bacteria) associations. Basic research focuses more on the biological mechanisms and potential therapeutic targets of *A. muciniphila*. The active components of *A. muciniphila* are still being clarified. Interestingly however, pasteurized *A. muciniphila*, live *A. muciniphila*, and even secreted proteins (e.g., Amuc_1100) and extracellular vesicles can regulate gut barrier function and/or the immune system by acting on different molecules. Regarding the effects of *A. muciniphila* on human health (e.g., “microbiota metabolites,” “immune system,” and “gut barrier function”), interested readers may wish to refer to previous detailed explorations of such topics (Yan et al., 2021; Rodrigues et al., 2022). The molecular mechanisms underlying these effects are an ongoing hot topic in the field. Recently, Bae et al. (2022) revealed that *A. muciniphila* induces immune cells to secrete specific cytokines *via* cell membrane phospholipids and resetting the activation threshold of dendritic cells, clarifying the molecular mechanism underlying *A. muciniphila-*mediated immune regulation *in vitro*. However, the molecular mechanisms underlying *A. muciniphila*–host interactions still require further research.
- The safety and efficacy of the clinical use of *A. muciniphila* (e.g., “double-blind”) (Depommier et al., 2019).

Among the top 25 keywords with the strongest citation bursts, “oxidative stress” showed increasing strength. Oxidative stress is caused by imbalances between intracellular reactive oxygen species and antioxidant defense systems (Papadia et al., 2008), and is considered an important risk factor for cardiovascular diseases, diabetes, and other diseases. Research on oxidative stress mainly focuses on three aspects: promoting oxidative stress, fighting oxidative stress, and balancing the oxidative and antioxidant systems. Several studies suggested that *A. muciniphila* may be associated with oxidative stress regulation (Yassour et al., 2016; Roshanravan et al., 2017; Mitsou et al., 2019; Wu et al., 2020; Zhang et al., 2020; Deng et al., 2021; Mesnage et al., 2021; Chen et al., 2022) and may promote oxidative stress resistance in various diseases (Cerro et al., 2022; Qian et al., 2022; Xia et al., 2022). Polyphenols decrease intestinal oxidative stress by inducing *A. muciniphila* growth (Anhê et al., 2015). The relationship between oxidative stress and *A. muciniphila* is an important topic for future research.

Our study has some limitations. Firstly, since it takes time for an article to achieve a certain number of citations, recent high-quality articles may not have been included, causing biased results. Secondly, there may be a time delay when exploring the research frontier. Finally, our analysis can only show the influence of the research content in the *A. muciniphila* research field, and cannot represent influences outside this field.

## Conclusion

We evaluated and quantified articles on *A. muciniphila* and visualized the hotspots and global research trends in this field. Over the past 19 years, publications on *A. muciniphila* have increased significantly in frequency, with China having the highest number of publications. De Vos and Willem was the most productive author and had the highest H-index, followed by Cani and Patrice. “Oxidative stress,” “diet,” “metformin,” “fecal microbiota transplantation,” “short-chain fatty acids,” “polyphenols,” and “microbiota metabolites” are some of the frequently used keywords in recent years. These keywords are potential hotspots for future research and require further exploration. Although studies consider *A. muciniphila* to be a beneficial probiotic and has potential in the treatment of many diseases, providing an in-depth analysis of the mechanisms underlying its role in promoting human health with respect to high-frequency diseases may improve the research status of *A. muciniphila*.

## Data availability statement

The original contributions presented in the study are included in the article/Supplementary material, further inquiries can be directed to the corresponding authors.

## Author contributions

YC, ZL, QL, and PW: conceptualization. HK, YW, XL, and SC: data curation. HK and ZL: writing—original draft preparation. ZL, HK, YW, SC, and YC: writing—review and editing. YC, QL, and PW: supervision. YC: funding acquisition. All authors contributed to the article and approved the submitted version.

## Funding

This work was supported by the Special Scientific Research Fund for National Natural Science Foundation, grant numbers 82070543 and 8177031240, and the National High Technology Research and Development Program of China, grant number 2021YFA0717001.

## Conflict of interest

The authors declare that the research was conducted in the absence of any commercial or financial relationships that could be construed as a potential conflict of interest.

## Publisher’s note

All claims expressed in this article are solely those of the authors and do not necessarily represent those of their affiliated organizations, or those of the publisher, the editors and the reviewers. Any product that may be evaluated in this article, or claim that may be made by its manufacturer, is not guaranteed or endorsed by the publisher.

## Footnotes

^1^ https://bibliometric.com/

^2^ www.figdraw.com

^3^ https://www.a-mansia.com/

## Abbreviations

ACI: Average citations per item
H-index: The Hirsch Index
IFs: Impact factors
JCR: Journal citation reports
MeSH: Medical subject headings
PNAS: Proceedings of the National Academy of Sciences of the United States of America
TLS: Total link strength
WoSCC: Web of Science Core Collection database
