# Supplementary material for: Global trends in Akkermansia muciniphila research: A bibliometric visualization
Source: Front Microbiol. 2022 Nov 10;13:1037708. doi: 10.3389/fmicb.2022.1037708 (PMC9685322; doi:10.3389/fmicb.2022.1037708)
Supplement: Supplementary file 1 [file Table_1.DOCX]

Supplementary Material

**Supplementary Table 1.** Top 10 most-cited references (2004-2022).

| **Rank** | **Title** | **Author** | **Year** | **Counts** | **Journal** | **IF (2021)** |
| --- | --- | --- | --- | --- | --- | --- |
| 1 | Cross-talk between *Akkermansia muciniphila* and intestinal epithelium controls diet-induced obesity | Everard A | 2013 | 319 | PNAS | 12.779 |
| 2 | *Akkermansia muciniphila* and improved metabolic health during a dietary intervention in obesity: relationship with gut microbiome richness and ecology | Dao MC | 2016 | 312 | GUT | 31.793 |
| 3 | A purified membrane protein from *Akkermansia muciniphila* or the pasteurized bacterium improves metabolism in obese and diabetic mice | Plovier H | 2017 | 305 | NAT MED | 87.241 |
| 4 | *Akkermansia muciniphila* and its role in regulating host functions | Derrien M | 2017 | 217 | MICROB PATHOGENESIS | 3.848 |
| 5 | *Akkermansia muciniphila* inversely correlates with the onset of inflammation, altered adipose tissue metabolism and metabolic disorders during obesity in mice | Schneeberger M | 2015 | 180 | SCI REP-UK | 4.996 |
| 6 | Supplementation with *Akkermansia muciniphila* in overweight and obese human volunteers: a proof-of-concept exploratory study | Depommier C | 2019 | 153 | NAT MED | 87.241 |
| 7 | An increase in the *Akkermansia* spp. population induced by metformin treatment improves glucose homeostasis in diet-induced obese mice | Shin NR | 2014 | 151 | GUT | 31.793 |
| 8 | Next-Generation Beneficial Microbes: The Case of *Akkermansia muciniphila* | Cani PD | 2017 | 146 | FRONT MICROBIOL | 6.064 |
| 9 | Dietary Polyphenols Promote Growth of the Gut Bacterium *Akkermansia muciniphila* and Attenuate High-Fat Diet–Induced Metabolic Syndrome | Roopchand DE | 2015 | 131 | DIABETES | 9.337 |
| 10 | A polyphenol-rich cranberry extract protects from diet-induced obesity, insulin resistance and intestinal inflammation in association with increased *Akkermansia* spp. population in the gut microbiota of mice | Anhe FF | 2015 | 128 | GUT | 31.793 |
